# Human health risk assessment of potentially toxic elements in the breast milk consumed by infants in Western Iran

**DOI:** 10.1038/s41598-023-33919-0

**Published:** 2023-04-24

**Authors:** Kiomars Sharafi, Samaneh Nakhaee, Nammam Ali Azadi, Borhan Mansouri, Saba Miri Kermanshahi, Maedeh Paknahad, Yazdan Habibi

**Affiliations:** 1 Research Center for Environmental Determinants of Health (RCEDH), Research Institute for Health, Kermanshah University of Medical Sciences, Kermanshah, Iran; 2 Medical Toxicology and Drug Abuse Research Center (MTDRC), Birjand University of Medical Sciences, Birjand, Iran; 3 Biostatistics Department, School of Public Health, Iran University of Medical Sciences, Tehran, Iran; 4 Substance Abuse Prevention Research Center, Research Institute for Health, Kermanshah University of Medical Sciences, Kermanshah, Iran; 5 Student Research Committee, Kermanshah University of Medical Sciences, Kermanshah, Iran

**Keywords:** Environmental sciences, Health care, Medical research

## Abstract

This study aimed to assess the human health risk of some toxic metals/metalloids [lead (Pb), mercury (Hg), cadmium (Cd), nickel (Ni), chromium (Cr), and arsenic (As)] on infants via consumption of the breast milk of women living in urban areas of Kermanshah city, west of Iran. After collecting milk samples, the carcinogenic and non-carcinogenic risk assessment as well as uncertainty analysis of toxic metal levels were carried out. The order of concentration of heavy metals/metalloids in the breast milk samples was Cr (41.07 ± 23.19) > Ni (19.25 ± 11.81) > Pb (11.5 ± 4.48) > As (1.96 ± 2.04) > Cd (.72 ± 0.42) > Hg (0.31 ± 0.26). The results revealed that the levels of Cr and Pb in the breast milk samples were exceeded the World Health Organization (WHO) tolerable daily intake. In the breast milk samples a high levels of one of the trace elements As, Cd, Cr, Pb, and Ni were observed (over 73%) and in 40% of them the levels of Cr, Pb, Cd, As, and Ni were all above WHO tolerable daily intake. Moreover, the As-related point assessment of target risk factor (THQ) was higher than the allowable limit only for 1-month-old male neonates and 2-month-old female neonates (THQ > 1). In addition, Cr-related THQ scores were higher at all age and gender groups (THQ > 1). In conclusion, our findings suggest a potential risk of some metals for infants via the consumption of mothers' breast milk.

## Introduction

Breastfeeding is essential to maintaine a good growth of the child^1^. The World Health Organization recommends that mother to breastfeed a child up to 6 months of age and then feeding with complementary foods for up to 2 years^2^. However, milk can transfer toxic elements from the mother to the baby via breastfeeding. The presence of these toxins does not necessarily cause health hazards in the infant, but since these hazards are biologically probable, they should be monitored^3^. The first stage of transmission of contaminants to the baby begins from the time of placement in the mother's womb and continues until breastfeeding^4^. Toxic heavy metals are one of the main chemical pollutants that enter the environment (water, soil, and air) naturally or through human activities and thay may enter the human body through inhalation, food, and skin transmission^5–7^ These heavy metals have toxic effects on human health and exposure to them can cause diffrent diseases^7^. Compared to adults, the infants are more sensitive to metal toxicity due to the rapid growth of body tissues, especially their nervous system^4^.

Among the heavy metals/metalloids, lead (Pb), cadmium (Cd), arsenic (As), and mercury (Hg) are of greater health importance to humans. These metals are not metabolized in the body which may lead to toxicity due to their accumulation in the tissues^8,9^. As one of the leading carcinogens, cadmium, transmit to humans body through water, food, inhalation, and smoking^10^. The average weekly Cd intake in European Food Safety Authority (EFSA) is set at 2.5 μg/kg^11^. Digestive absorption of Cd during lactation (37%) is higher than in adulthood (5%). Therefore, maternal exposure to Cd, even at low environmental concentrations, can lead to health risks, especially neurological changes and deformities in the infant's bones^10,12^.

Another heavy metal, lead, can also enter the body through water, food, inhalation, and the skin pathway^5,13^. If Pb consumed orally, about 5–10% of Pb is absorbed. This increases to 45% in children^14^. The neurological effects of Pb in children include movement disorders and behavioral problems^12^. The recommended daily intake of Pb for infants is 5 μg/kg^15^. The International Agency for Research on Cancer has placed Pb in the second category of carcinogenic compounds^16^.

Arsenic is also a metalloid that can be transmitted through inhalation of As-contaminated air, food contaminated with As-containing pesticides as well as contaminated water^17^. As is known to be a carcinogen that can cause skin, liver, kidney, lung, and bladder cancers and adversely affects the nervous, cardiovascular and respiratory systems^18^. The International Agency for Research on Cancer has placed As in the first group of carcinogenic compounds^16^. In 2010, the Joint FAO/WHO Expert Committee on Food Additives (JECFA) set a weekly intake of 1.2 μg/kg for As^11^.

Humans are exposed to organic, inorganic, and metallic Hg mainly through the consumption of fish, medicinal and cosmetic compounds, and dental amalgam^19^. Mineral and elemental Hg are excreted mainly in the urine. Significant amounts of Hg are also excreted via sweat, tears, and breast milk^20^. JECFAsuggests a permissible weekly intake of 1.6 μg/kg for Hg^21^. Fetuses and infants are exposed to methylmercury, which is the most toxic form of Hg, that determines 5 μg/kg for total Hg^22^. Some studies have reported the presence of toxic metals in human milk around the world^11,23–27^.

On the contrary, the low concentrations of metals in human milk were determined in some reports^28–30^. There is little research available on the health risk of toxic metals/metalloids on infants transmitted via the breast milk using Iranian population. Therefore, this study aimed to measure the concentrations levels as well as the human health risk (non-carcinogenic and carcinogenic risk assessment) of six heavy metals/metalloids (Pb, Hg, Cd, Ni, Cr, and As) on infants via the breast milk of women in Kermanshah city, located in the west of Iran. We hypothesize that elevated heavy metal concentrations may have non-carcinogenic and carcinogenic risks for neonates.

## Materials and methods

### Study population

This study was a cross-sectional study in which after obtaining informed consent from participants, 100 samples were collected from lactating women living in urban areas of Kermanshah city during September to December 2021. This research was funded by Kermanshah University of Medical Sciences (ethic code: IR.KUMS.REC.1399.222). Participants were healthy breastfeeding mothers who were primiparous and breastfeed only one child after a normal pregnancy with no complications. They were residence of Kermanshah for at least five years. Mothers with chronic pre-pregnancy diseases (cardiac or autoimmune diseases) and also those living in areas with known emissions or suspected to the elevated levels of pollutants were excluded^6^.

### Breast milk sample collection

The health center’s staff carried out all sampling procedures according to the study protocols. After the admission of lactating women to the study, 5 to 10 ml of milk was collected during morning hours by manual expression from each person. Mothers were instructed to wash their hands and chest areas before collecting samples. Collected samples were labeled inside sterile polyethylene tubes. The milk samples were then kept at − 20 °C until further analyses^31^.

### Drinking water

In this study, the volume of water used was 5 cc. The sample locations were in Kermanshah city and similarly at the same time as the samples of mothers’ milk. We used sterilized plastic bottles that had already been washed with distilled water and 20% Nitric acid (HNO_3_). For sampling, the tap water was opened for few seconds to make sure the municipal water network is collected. To prevent adsorption and crystallization of the trace element, water samples were then filtered using a 0.45 mm Whatman’s membrane and acidified with 3 ml of nitric acid (HNO_3_, 65%, Merck, Germany)^32^. Samples were then transferred to cool, dark containers and kept at 4 °C until Inductively coupled plasma mass spectrometry (ICP-MS) analyses.

### Heavy metals/metalloids in water and milk measurements

To prepare milk samples for digestion, they exposed to the room temperature. For each sample, 1.0 ml of milk was placed in a test tube and then 5 ml of nitric acid (HNO_3_, 65%, Merck, Germany) was added to the tube. The sample was placed at room temperature overnight to digest slowly. At the next day, 2 ml of 35% hydrogen peroxide (H_2_O_2_) was added to the tube and placed inside the water bath (TW12, Julabo GmbH, Germany) for 6 h or until the solution was clear at 98 °C. The samples were diluted with ultrapure water (18.2 MΩ-cm at 25 °C, Fistreem, WSC044, UK) up to 25 mL^23^. Concentrations of As, Cd, Cr, Hg, Ni, and Pb in the milk and water samples were measured with Inductively Coupled Plasma Mass Spectrometry (ICP-MS, Agilent 7900, Santa Clara, CA, USA). The recovery of As, Cd, Cr, Hg, Ni and Pb were 97, 102, 96, 98 and 99% respectively.

### Human health risk assessment

Considering that the contaminants in breast milk, especially toxic metals, can cause acute and chronic health effects on infants, the health risk assessment of the toxic metals was carried out.

### Non-Carcinogenic risk assessment

The non-carcinogenic health risk after exposure to a toxic element was determined using the ratio of the estimated daily intake (EDI) of the element over the target hazard quotient (THQ) of that element, that is

$$\mathrm{THQ}=\frac{EDI}{RfD},$$

where the oral reference dose (*RfD*) of trace elements As, Cd, Pb, Ni, Hg, and Cr were 0.0003, 0.001, 0.0035, 0.02, 0.0001 and 0.003 mg/kg respectively^33–35^ and EDI was estimated using following equation:

$$\mathrm{EDI}=\frac{DCBM\times CHM}{BW},$$

where CHM denotes the concentration of heavy metals as reported in Table 1, BW is the neonatal body weight, and DCBM represents the daily consumption of breast milk. BW and DCBM by sex and age groups is given in Table S1 at Supplementary Materials.

**Table 1:** The heavy metals concentration of breast milk.

Heavy metals	Descriptive parameters (µg L^−1^)				WHO	
	Mean	SD	Min	Max	TDI	Percentage of infats that Exceeded the MTL (µg L^−1^)
As	1.96	2.04	0.06	11.76	0.3	75%
Cd	0.72	0.42	0.07	2.11	0.4	74%
Hg	0.31	0.26	0.05	1.12	0.7	9%
Pb	11.5	4.48	4.11	21.74	3.6	100%
Cr	41.07	23.19	11.02	128.72	0.9	100%
Ni	19.25	11.81	0.10	66.98	12	72%

*TDI* tolerable daily intake, *MTL* maximum tolerable limit.

The total target hazard quotients (TTHQ) was calculated by adding up the target hazard quotients of each trace element.

$$\mathrm{TTHQ}={THQ}_{As}+{THQ}_{Pb}+\dots +{THQ}_{Cr}.$$

According to USEPA, the acceptable value is 1 for THQ (and TTHQ).

### Carcinogenic risk assessment

The total carcinogenic risk from various metals was estimated using

$$\mathrm{TCR}={ILCR}_{As}+{ILCR}_{Pb}+\dots +{ILCR}_{Cr},$$

where incremental lifetime cancer risk (ILCR) for a particular metal was obtained by EDI × CSF^4,30^. The cancer sloop factor (CSF) for As, Cd, Pb, Ni, and Cr were defined as 1.5, 6.3, 0.0085, 0.91, and 0.5, respectively. A carcinogenic risk is acceptable (tolerable) if TCR ≤ 10.

### Uncertainty analysis approach

Since CHM, BW, and DCBM parameters vary between genders and trace elements (Supplementary Material, Table S1), the average number of these parameters is used in risk assessments^36^. As a consequence, the EDI is estimated as a point and may not reflect properly the actual exposure of the study population to the heavy metals^11,37^. Inappropriate estimation of EDI will affect THQ, TTHQ, ILCR, and also TCR parameters. Therefore, the uncertainty analysis using the Monte-Carlo simulation technique with 100,000 iterations was peformed using the Oracle Crystal Ball EXCEL plug-in (Ver.11.1.2.4). This allows us to vary HMC, BW, and DCBM parameters (according to Table S1) and estimate the uncertainty bound (5th to 95th percentile) for TTHQ and TCR.

### Statistical analysis

The Excel and SPSS software (version 18) was used to analyze the data. Heavy metal concentrations were presented as mean and standard deviation. The Pearson correlation test was also used to investigate the relationship between heavy metal concentrations in drinking water samples and milk. A significance level of 5% was considered.

### Compliance with ethical standards

This study was conducted by the World Medical Association Declaration of Helsinki. This study was approved by the Research and Ethics Committee of Kermanshah University of Medical Sciences (IR.KUMS.REC.1399.222).The informed consent was obtained from participants/ parents/legal guardian. We confirm that all methods were carried out in accordance with relevant guidelines and regulations.

## Results

### Heavy metals/metalloids in drinking water and milk samples

**Figure 1:**
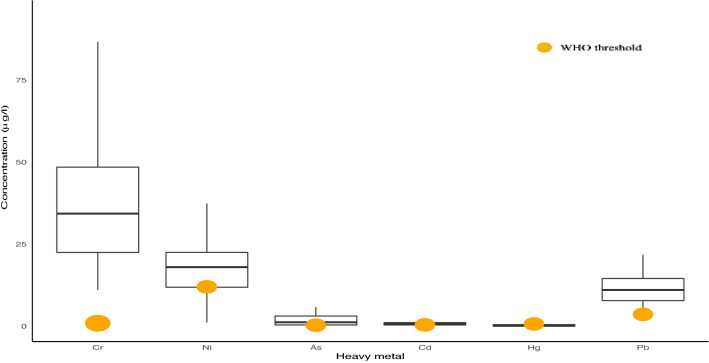
Box-plot of heavy metal concentration levels in breast milk of participants. The bold point in orange shows WHO TDI.

The average concentrations of heavy metals/metalloids in milk and drinking water samples are presented in Table 1 and Supplementary Material, Table S2, respectively. The order of accumulation of heavy metals/metalloids in breast milk samples was Cr (mean = 41.07 µg L^−1^) > Ni (mean = 19.25 µg L^−1^) > Pb (mean = 11.5 µg L^−1^) > As (mean = 1.96 µg L^−1^) > Cd (mean = 0.72 µg L^−1^) > Hg (mean = 0.31 µg L^−1^). In drinking water concentration levels followed a decreasing order as Ni (mean = 16 µg L^−1^) > As (mean = 7.9 µg L^−1^) > Cr (mean = 6.2 µg L^−1^) > Pb (mean = 3.3 µg L^−1^) > Cd (mean = 0.1 µg L^−1^) > Hg (mean = 0.5 µg L^−1^). The box plot of heavy metal/metalloids concentration levels in breast milk and drinking water with corresponding WHO and EPA threshold values for each metal are presented by Fig. 1 and Supplementary Material, Figure S1 respectively. It can be seen clearly that the concentrations of Cr and Pb in all breast milk samples were well above WHO's tolerable daily intake (0.9 µg/L for Cr and 3.6 µg/L for Pb). The percentage of breast milk samples exceeded WHO tolerable daily intake of As (0.3 µg/L) was 75%. Moreover, 73% and 74% of breast milk samples exceeded TDI_WHO_ for Cd (0.4 µg/L) and Ni (12 µg/L) respectively. Another notable point to mention is that in 40% of breast milk samples the levels of Cr, Pb, Cd, As, and Ni were all above WHO thresholds.

The mean concentration of Hg was 0.31 µg L^−1^ which was below WHO Maximum Tolerable Limit (MTL) (0.72 µg L^−1^) (only 9% of subjects exceeded WHO MTL). Heavy metal concentration levels in drinking water, presented by Supplementary Material, Fig. S1, were well below both WHO and EPA guideline values; All Cr, Pb, Ni, Cd, and Hg concentration values (10 of 10 concentrations values) were below the WHO standard threshold. Two values of As concentration levels (20%) were above the WHO recommended standard point.

### Correlation of heavy metals/metalloids in water with breast milk

We also investigate the potential association between concentration levels of heavy metals/metalloids in breast milk and drinking water using Pearson correlation. Since only 10 water samples were obtained from the city water supply, but we had 100 breast milk samples available, the correlation was estimated using 10 water samples and 10 breast milk samples randomly selected out of 100 available samples. This procedure was repeated 100,000 times and each time the correlation and its corresponding p-value were obtained for each metal. The correlation between the Cr levels in breast milk and drinking water was only significant for 4963 samples (4.9%), for Ni at (4.7%), As (4.7%), Cd (4.9%), Hg (4.4%), and Pb (5.1%). We may therefore conclude that there was no strong evidence to claim the association between heavy metal levels in breast milk and drinking water.

### Health risk assessment

**Figure 2:**
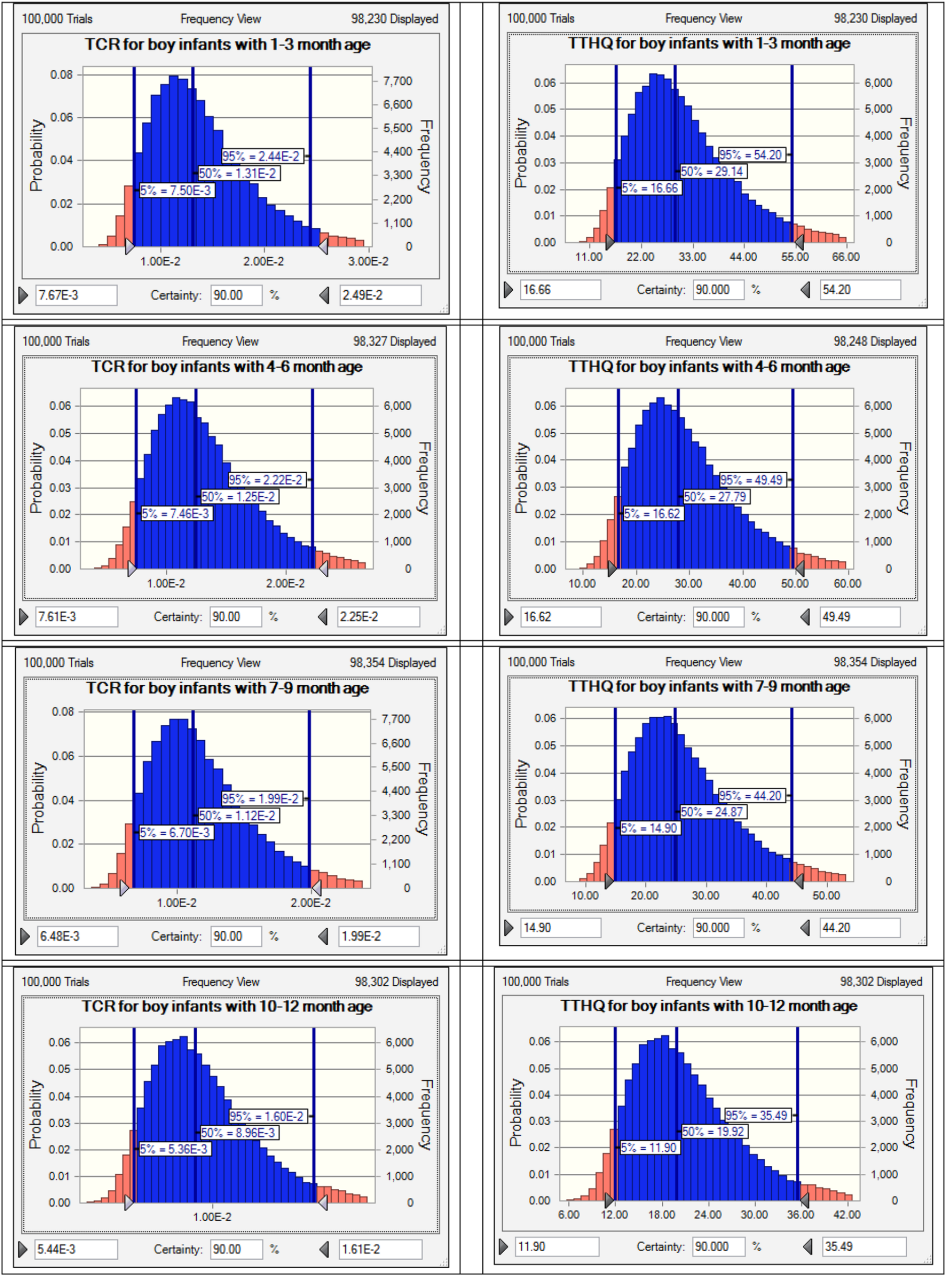
the uncertainty analysis for TTHQ and TCR related to toxic metals of breast milk for boy infants with different age.

**Figure 3:**
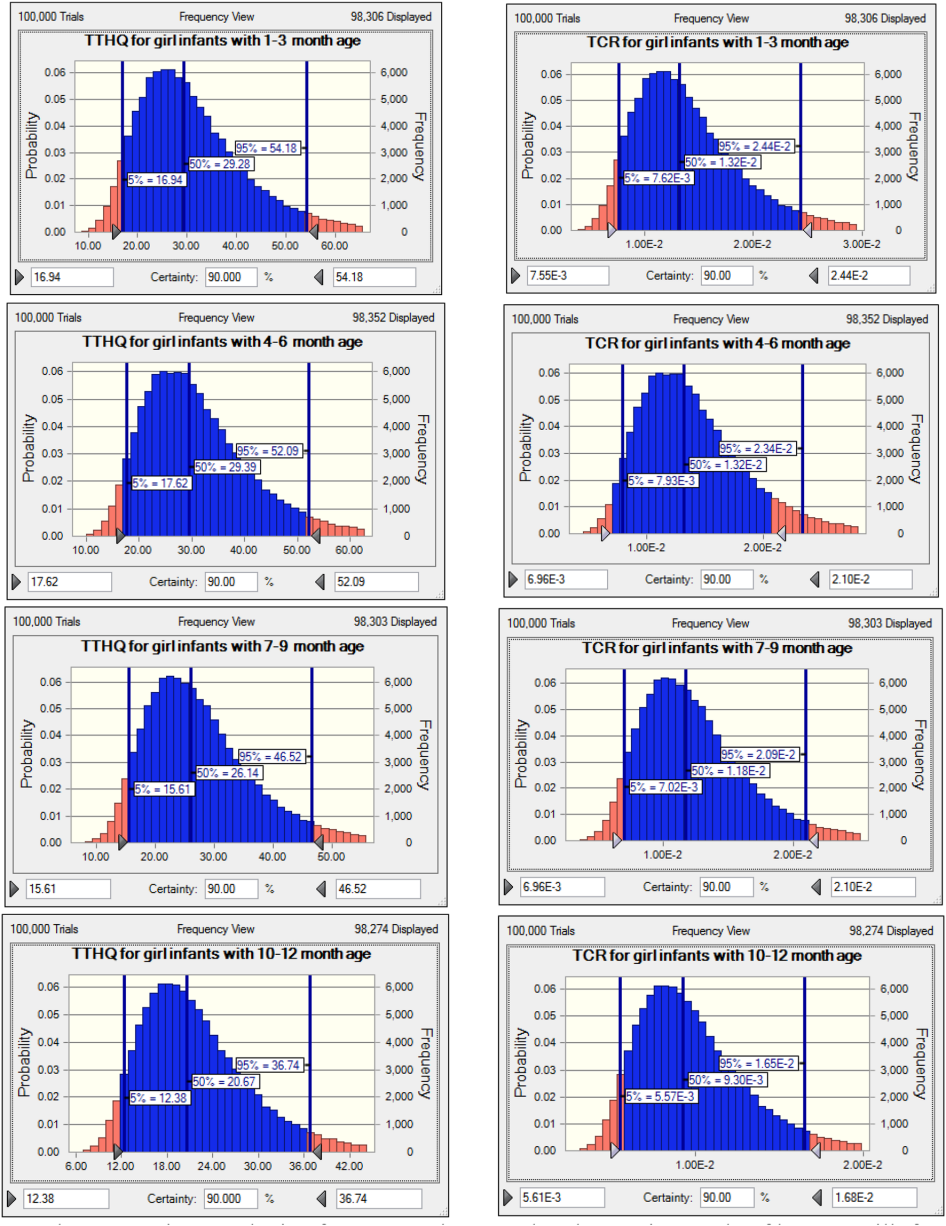
The uncertainty analysis of TTHQ and TCR related to toxic metals of breast milk for girl infants with different age.

The results of the present study showed that the As-related point assessment of THQ was higher than the allowable limit for 1-month-old male neonates and 2-month-old female neonates (THQ = 1). This factor was lower for other age groups in terms of As metal (Table 2). In addition, Cr-related THQ scores were higher for all age groups and in both neonatal groups, while THQ was lower for other metals in all age groups and both neonates (Table 2). The point estimation of the ILCR parameter for all carcinogenic metals shows that this parameter was higher than the allowable limit for all age groups and both sexes of infants (ILCR = 1 × 10^−4^) (Table 2). In this study, to ensure the total amount of non-carcinogenic and carcinogenic effects of different metals, TTHQ and TCR factors by uncertainty analysis method for four age groups of infants including 3–1, 6–4, 7–9, and 10–12 months and it was calculated for both sexes (Figs. 2 and 3). The results of uncertainty analysis showed that TCR related to carcinogenic effect and TTHQ related to cumulative non-carcinogenic effect related to different metals in breast milk for all the above age groups and both sexes of infants, with a large difference from the acceptable level (TTHQ = 1 and TCR = 10^−4^) were higher (Figs. 2 and 3).

**Table 2:** The EDI, THQ, and ILCR related to toxic metals of breast milk for infants of different age.

Heavy metals	Age (Month)	EDI (mg/kg.bw-d)		THQ		ILCR	
		Boy	Girl	Boy	Girl	Boy	Girl
As	1	3.2E − 04	3.0E − 04	1.06	0.99	4.8E − 04	4.4E − 04
	2	3.0E − 04	3.1E − 04	0.98	1.04	4.4E − 04	4.7E − 04
	3	2.8E − 04	3.0E − 04	0.94	1.00	4.2E − 04	4.5E − 04
	4–6	2.3E − 04	2.5E − 04	0.77	0.82	3.5E − 04	3.7E − 04
	7	2.2E − 04	2.3E − 04	0.72	0.76	3.2E − 04	3.4E − 04
	8	2.1E − 04	2.2E − 04	0.70	0.73	3.1E − 04	3.3E − 04
	9	2.0E − 04	2.1E − 04	0.67	0.70	3.0E − 04	3.2E − 04
	10–12	1.7E − 04	1.7E − 04	0.56	0.58	2.5E − 04	2.6E − 04
Cd	1	1.2E − 04	1.1E − 04	0.12	0.11	7.4E − 04	6.9E − 04
	2	1.1E − 04	1.1E − 04	0.11	0.11	6.8E − 04	7.2E − 04
	3	1.0E − 04	1.1E − 04	0.10	0.11	6.5E − 04	6.9E − 04
	4–6	8.5E − 05	9.1E − 05	0.09	0.09	5.4E − 04	5.7E − 04
	7	8.0E − 05	8.4E − 05	0.08	0.08	5.0E − 04	5.3E − 04
	8	7.7E − 05	8.0E − 05	0.08	0.08	4.8E − 04	5.1E − 04
	9	7.4E − 05	7.8E − 05	0.07	0.08	4.7E − 04	4.9E − 04
	10–12	6.1E − 05	6.4E − 05	0.06	0.06	3.9E − 04	4.0E − 04
Hg	1	5.0E − 05	4.7E − 05	0.50	0.47	–	–
	2	4.7E − 05	4.9E − 05	0.47	0.49	–	–
	3	4.4E − 05	4.7E − 05	0.44	0.47	–	–
	4–6	3.7E − 05	3.9E − 05	0.37	0.39	–	–
	7	3.4E − 05	3.6E − 05	0.34	0.36	–	–
	8	3.3E − 05	3.5E − 05	0.33	0.35	–	–
	9	3.2E − 05	3.3E − 05	0.32	0.33	–	–
	10–12	2.6E − 05	2.8E − 05	0.26	0.28	–	–
Pb	1	1.9E − 03	1.7E − 03	0.53	0.50	1.6E − 05	1.5E − 05
	2	1.7E − 03	1.8E − 03	0.49	0.52	1.5E − 05	1.6E − 05
	3	1.6E − 03	1.8E − 03	0.47	0.50	1.4E − 05	1.5E − 05
	4–6	1.4E − 03	1.4E − 03	0.39	0.41	1.2E − 05	1.2E − 05
	7	1.3E − 03	1.3E − 03	0.36	0.38	1.1E − 05	1.1E − 05
	8	1.2E − 03	1.3E − 03	0.35	0.37	1.0E − 05	1.1E − 05
	9	1.2E − 03	1.2E − 03	0.34	0.35	1.0E − 05	1.1E − 05
	10–12	9.8E − 04	1.0E − 03	0.28	0.29	8.3E − 06	8.7E − 06
Cr	1	6.7E − 03	6.2E − 03	2.23	2.07	3.3E − 03	3.1E − 03
	2	6.2E − 03	6.5E − 03	2.06	2.18	3.1E − 03	3.3E − 03
	3	5.9E − 03	6.3E − 03	1.96	2.09	2.9E − 03	3.1E − 03
	4–6	4.9E − 03	5.2E − 03	1.62	1.72	2.4E − 03	2.6E − 03
	7	4.5E − 03	4.8E − 03	1.51	1.59	2.3E − 03	2.4E − 03
	8	4.4E − 03	4.6E − 03	1.46	1.53	2.2E − 03	2.3E − 03
	9	4.2E − 03	4.4E − 03	1.41	1.48	2.1E − 03	2.2E − 03
	10–12	3.5E − 03	3.6E − 03	1.17	1.21	1.8E − 03	1.8E − 03
Ni	1	3.1E − 03	2.9E − 03	0.16	0.15	2.8E − 03	2.7E − 03
	2	2.9E − 03	3.1E − 03	0.14	0.15	2.6E − 03	2.8E − 03
	3	2.8E − 03	2.9E − 03	0.14	0.15	2.5E − 03	2.7E − 03
	4–6	2.3E − 03	2.4E − 03	0.11	0.12	2.1E − 03	2.2E − 03
	7	2.1E − 03	2.2E − 03	0.11	0.11	1.9E − 03	2.0E − 03
	8	2.1E − 03	2.2E − 03	0.10	0.11	1.9E − 03	2.0E − 03
	9	2.0E − 03	2.1E − 03	0.10	0.10	1.8E − 03	1.9E − 03
	10–12	1.6E − 03	1.7E − 03	0.08	0.09	1.5E − 03	1.6E − 03

## Discussion

### Heavy metals/metalloids in milk samples

In this study, we characterized the concentrations of six heavy metals/metalloids (Pb, Hg, Cd, Ni, Cr, and As) in the breast milk samples of women living in urban areas of Kermanshah city. We found that the concentration levels of Cr and Pb in the breast milk samples were above WHO tolerable daily intake. Apart from these two trace elements, we also found a high levels of one of the trace elements As, Cd, and Ni (over 73%) in the breast milk samples. In 40% of breat milk samples, the levels of Cr, Pb, Cd, As, and Ni were all above WHO tolerable daily intake.

A summary of literature findings on concentration levels of toxic metals in the breast milk samples is presented in Table 3. The consistent presence of the selected toxic metals indicates the risk of contaminantion in the breast milk of lactating mothers which may have negative effects on the child's health. Infants are known more vulnerable if exposed to neurotoxins. Infants have a higher risk of heavy metal accumulation due to lower excretion rate and body weight, as well as a low immunity state^12^. It is also known that the toxicant uptake in the gastrointestinal system is greater for infants and newborns^38^. The higher levels of Pb and Cr and other metals in breast milk indicate exposure to various sources of these metals in Kermanshah province. Previous researches confirmed the presence of these toxic metals in soil^39,40^, street dust^41^, air^42^, rice^43^, vegetables ^44^, and cosmetics^45^ in this region.

**Table 3:** Summary of literature findings of arsenic, lead, chromium, nickel, and cadmium levels (µg L^-1^) in breast milk.

Study	Heavy metals						Country
	Cd	Pb	Hg	As	Ni	Cr	
Present study	0.72	11.5	0.31	1.96	19.25	41.07	Iran
Al-Saleh 2021^19^	1.66 ± 0.61	48.1 ± 24.0	2.20 ± 0.46	–	–	–	Saudi Arabia
Ekeanyanwu et al. 2020^28^	29 ± 13	38 ± 13	–*	–*	–	19 ± 11	Nigeria
Khan et al. 2018^27^	52 (2–301)**	95 (9–440)**	0.61 (0.2–3.98)**	0.50 (0.09–1.24)**	–	–	Pakistan
Tahboub et al. 2021^31^	6.31 ± 7.0	77.4 ± 68.2	–	31.4 ± 24.0	302 ± 453	132 ± 92	Jordan
Kunter et al. 2017^29^	0.45 ± 0.23	1.19 ± 1.53	0 ± 0.23	0.73 ± 0.58	–	–	Cyprus
Winiarska-Mieczan 201 ^12^	2.11 ± 2.11	6.33 ± 4.61	–	–	–	–	Poland
Gurbay et al. 2012^30^	–*	391.4 ± 269.0	–	–*	43.9 ± 33.8	–	Turkey
Nazlican et al. 2022^64^	0.37	12.12	2.60	1.64	–	8.25	Turkey
Almeida et al. 2008^65^	–	0.94 ± 1.05	–	5.8 ± 1.1	5.8 ± 1.8	–	Portugal
Park et al. 2018^25^	0.72	13.3 ± 26.0	1.19 ± 1.24				Korea

*below detection limits.

**data presented mean (range).

This study showed higher Pb levels in breast milk compared to similar studies conducted in Iran. For example, Rahimi et al., Goudarzi et al., and Samiee presented mean Pb levels of 2.44, 7.11, and 75 μg/L, respectively^23,46,47^, these observations are comparable to previous literature in other countries that reported a similarly elevated level of Pb in breast milk over the lactation course^25,27,28,38^. Park et al. in the CHECK cohort study demonstrated that Pb levels of some breast milk samples were above the normal values suggested by WHO. The percentage of infants exceeding the standard value of Pb was 71% and 56% for 15-day, and 30-day-old infants, respectively^25^. Another study reported a higher reference value of 95% (RV 95) compared to the WHO/ International Atomic Energy Agency (IAEA) reference range for Pb. All the 203 lactating mothers in their study had a Pb concentration above the WHO/IAEA reference value in breast milk^19^. Another study on 100 lactating mothers in Hamedan showed that 94% of the breast milk samples exceeded the WHO standard range for Pb^23^. Lead exposure in the general population is via contaminated drinking water, food, and the inhalation of Pb particles. Morever, both active and passive smoking can also contribute to elevate Pb levels^19^. Breastfeeding can be associated with increased bone resorption in response to the calcium needs of breastfed infants^25^. In addition, during the mobilization of calcium in the mother’s bones in the second half of pregnancy for fetal skeletal growth, Pb enters the mother's bloodstream^23^. The proportion of infants who have exceeded the reference ranges of studied metals, especially for Pb, is alarming. Lead can cause neurological complications in children, including movement and behavioral problems^12^. A study suggestes that neurodevelopment and walking age are negatively affected in infants living near smelters and fishing villages and therefore are exposed to high levels of Pb via breast milk^48^. Low intelligence quotient (IQ) was observed in infants who were breastfed with Pb-contaminated milk^49^. Longer-term follow-up studies for infants exposed to high Pb levels are necessary to understand the consequences of neurodevelopment in the early life stage.

Chromium (Cr) is a heavy metal that is naturally distributed in the environment and some industrial activities can release large amounts of Cr compounds of different forms in the living environment^23^. The Cr and As contents of breast milk of 100 lactating mothers in hamedan, Iran were above the limit of detection (LOD) in 19% and 76% of samples, respectively. The breast milk Cr content in 74.8% of samples were above the safe range recommended by the WHO^23^. An study using a Nigerian population showed that the levels of the Cr in the breast mike was higher than the allowable limit^28^. Postnatal feeding infants through hexavalent Cr-contaminated breast milk can expose them to excessive oxidative stress^50^.

Cadmium (Cd) is another toxic element that has unknown function in the human body^28^. The primary sources of Cd exposure in the general population are diet and smoking. It can exert its toxic effects on the kidney, respiratory and skeletal systems^51^. There is a potent association between Cd concentration in breast milk and the secretion of calcium in breast milk^52^. This indicates that there may not be enough calcium in breast milk due to maternal exposure to Cd. The concentration of Cd in breast milk observed in the current study is generally comparable to the previous studies^28,31^. Feizi el al. study showed that 87% of the cosmetic samples exceeded the WHO limit for Cd levels in Pakistan^27^. Al-Saleh reported that the reference value in Saudi Arabia was higher than the WHO/IAEA reference range of less than 1 μg/L for Cd in breast milk in normal condition. Of the 203 mothers who participated in their study, 190 (93.6%) mothers exceeded the WHO/IAEA limits for Cd in breast milk^19^.

As, a well-documented toxic metalloid, is particularly dangerous for infants and young children who have chronic exposure, associated with low IQ, poor mental function, cognitive decline, and cancer^53^. In a study on Iranian women, Samiee et. al. (2019) reported that the concentration levels of As in 19% of breast milk samples was higher than the WHO recommended level for As^23^. Released reports from Taiwan and Sweden, both of which found milk As concentrations less than 1 μg/L^38,54^. The mechanism of excretion for As through breast milk is not known. Some findings indicate that inorganic As are not easily transmitted through breast milk. A study in Bangladesh (2008), claimed that even among women exposed to the high levels of As via groundwater the levels of inorganic As in their milk was relatively low^55^. This is in agreement with our finding of no strong evidence for the association between heavy metal/metalloid concentration levels in breast milk and drinking water.

The current study showed that the mean concentration of Hg was 0.31 µg L^−1^ which was below the WHO Maximum Tolerable Limit (only 9% of subjects exceeded WHO MTL). A global and regional review of trends in total mercury concentrations in human breast milk and whole blood suggested that South America, followed by Africa and Asia had the highest concentrations of total mercury while Europe and North America had the lowest values in the selected biological matrices^56^. In the countryside of Tehran, Tabriz, and Noushahr, Iran, human milk samples gave a mean mercury level of 0.12, 0.86, and 0.15 g/L, respectively. When compared to the WHO-recommended limit, only 3.7% of samples had mercury concentrations higher than normal^57^. A study in Saudi Arabia detailed that mercury levels in human breast milk were 2.19 μg l^_1^ and 4.15 μg l^_1^ for Al-Ehssa and Riyadh residents, respectively^58^. In 68 colostrum samples collected in Taiwan, the geometric mean mercury level was 2.03 μg l^_1^(ranged from: 0.24–9.45 μg l^_1^)^59^.

In this study, 72% of subjects exceeded the maximum tolerable limit for concentration levels of Ni in breast milk. Ni is known to be toxic to several body systems. It is defined as a nephrotoxic, neurotoxic, hepatotoxic, and carcinogenic agent and it can be also toxic to the pulmonary and reproductive systems^30^. International Agency for Research on Cancer (IARC) classified Ni compounds as Group 1agents^30^. Gürbay reported mean nickel concentrations in 64 breast milk samples collected in Turkey as 43.94 ± 33.82 μg/l. they reported that nickel Levels were also partly exceeded the recommended provisional tolerable weekly dietary intake (PTWI) limits^30^. In a study from Yazd, Iran, the mean Ni levels in breast milk samples on days 3–5, 16, and 30 after delivery were 47.3 ± 7.40, 49.9 ± 8.05, and 54.8 ± 7.38 μg/l, respectively. Also, its concentration in more than 86% of samples was higher than the permissible limits^60^. Osmar (2014) also found that breast milk from 48 Brazilian women living in Minas Gerais contained lower levels of nickel than WHO reference levels^61^. Consuming food, drinking water, and coming into direct contact with nickel-containing items like coins, stainless steel, and jewelry are all possible explanations for the higher concentration of nickel in breast milk. Due to the high levels of nickel in some foods like coffee, tea, chocolate, nuts, potatoes, cabbage, oatmeal, and spinach, these foods can be a major source of nickel exposure^30^.

### Health risk assessment

Our results showed that AS-related point assessment of THQ was higher than the allowable limit only for 1-month-old male neonates and 2-month-old female neonates. In addition, Cr-related THQ scores were higher at all age groups and in both neonatal groups.

In line with our results, Samiee et al. reported unacceptable non-cancer health risk levels or hazard quotient (HQ) for Pb and As in 61% and 10% of breast milk samples, respectively^23^.

Another study in Saudi Arabia showed that HQ of Cd and Hg was more than 1 indicating exposure to these toxic metals through breast milk feeding has noncarcinogenic effects. They also reported a neurotoxic risk of Pb for breastfed infants^19^. Many changes in toxic metal concentrations and their health effects, may be due to differences in environmental exposures and dietary differences among populations. In our study, the point estimation of the ILCR parameter for all carcinogenic metals showed that this parameter was higher than the acceptable limits for both gender at all age groups. Moreover, our study revealed that the risk of trace elements in younger infants was higher than that of in older infants. This outcome may be due to the greater vulnerability of the growing nervous system to the toxic effects of metals.

In the study of Al-Saleh et al. (2021) the mean CR for Pb from breast milk consumption was 3.7 × 10^−5^ with two values higher than the US EPA acceptable level of 1 × 10^−4^. In addition, the CR was significantly higher in younger infants (< 6 months) (4.7 × 10^−5^) than in infants aged 6 to 12 months (3.1 × 10^−5^)^19^. Agency for Research on Cancer (IARC) has defined Pb and its inorganic compounds as possible human carcinogens (group 2B and 2A, respectively). It also classified As as Group 1 and Cd as class B1^62^. Hexavalent Cr is a known carcinogen strongly correlated with lung cancer. It has also been reported as having a detrimental effect on embryonic development for both Cr (III) and Cr (IV). Beaumont et al. (2008) conducted a study in Liaoning Province, China, and reported an increased risk of cancer following the consumption of Cr (VI) via drinking water. In this regard, an increased risk of gastric cancer was shown, but some evidence also indicated an increased risk of lung cancer^63^. Therefore, the potential carcinogenic risk to infants through breastfeeding should not be ignored. Our results highlight the need to monitor the concentration of toxic metals in breast milk in the first 6 months of life as it is the primary food source for infants. However, this should be kept in mind that since the risk assessment approaches assume 100% of the toxic elements are absorbed and biologically effective, they are inherently noisy and may underestimate or overestimate the risk prediction.

## Limitation

Several limitations should be considered in this study. The volume of milk consumption by infants was calculated based on the published data. Longitudinal measurements of the metal concentration levels during the lactation course didn’t carried out in this study. No information was obtained about the dietary habits of mothers. In the current study, we assessed the risk of toxic heavy metals only from breast milk consumption and all other possible related sources were not assessed. The health risk assessment does not consider interactions between metals as a mixture, and therefore, if the interactions are more than additive, the health risk may be underestimated or if the interactions are less than additives, it may be overestimated.

## Conclusion

In this study, we assessed the concentrations of six heavy metals/metalloids (Pb, Hg, Cd, Ni, Cr, and As) in the breast milk samples. We found that in all samples the concentration levels of both Cr and Pb were well above WHO tolerable daily intake. We also found a high levels of one of the trace elements As, Cd, and Ni (over 73%) in the breast milk samples. In 40% of breat milk samples, the levels of Cr, Pb, Cd, As, and Ni were all above WHO tolerable daily intake. We also found no strong evidence to establish an association between heavy metal/metalloid concentration levels in breast milk and drinking water. Our results suggest that there may be a potential risk of some studied metals for infants via the consumption of mothers' breast milk.

Our results demonstrated that the As-related point assessment of THQ was higher than the allowable limit only for 1-month-old male neonates and 2-month-old female neonates. In addition, Cr-related THQ scores were higher for all age groups and in both neonatal groups. The point estimation of the ILCR parameter for all carcinogenic metals showed that this parameter was higher than the allowable limit for all age groups and both sexes of infants. Also, our study revealed that the risk of metals in younger infants was higher than in older infants. The observations of the current study may not represent the nationwide status of breast milk contamination with heavy metals. However, our study can add information about the range of studied toxic metal concentrations. Given the benefits of breast milk for the health and nutrition of the infant, this study should be repeated periodically with a larger sample size and in other parts of the world. This study is an update on the current status of toxic metal contamination in the breast milk of women in western Iran. According to our results, it is suggested that routine monitoring of potential sources of heavy metal contaminations in mothers and their infants should be performed to explore the contributing factors and develop appropriate interventions for mitigating the exposure.

## Supplementary Information

Supplementary Information 1.

Supplementary Information 2.

## Data Availability

The datasets used and analyzed during the current research are available from the corresponding author on request.
