# Supplementary material for: Human health risk assessment of potentially toxic elements in the breast milk consumed by infants in Western Iran
Source: Sci Rep. 2023 Apr 24;13:6656. doi: 10.1038/s41598-023-33919-0 (PMC10126154; doi:10.1038/s41598-023-33919-0)
Supplement: Supplementary file 1 — Supplementary Information 1. [file 41598_2023_33919_MOESM1_ESM.docx]

# Highlights

- The order of levels of toxic metals in breast milk samples was Cr > Ni > Pb > As > Cd > Hg.
- Levels of Cr and Pb in the breastmilk of all participants were well above WHO tolerable daily intake.
- There was no a strong evidence the association between heavy metal levels in breast-milk and drinking water.
- As and Cr-related point assessment of THQ was higher than the allowable limit for both neonatal groups
