# Supplementary material for: Human health risk assessment of potentially toxic elements in the breast milk consumed by infants in Western Iran
Source: Sci Rep. 2023 Apr 24;13:6656. doi: 10.1038/s41598-023-33919-0 (PMC10126154; doi:10.1038/s41598-023-33919-0)
Supplement: Supplementary file 2 — Supplementary Information 2. [file 41598_2023_33919_MOESM2_ESM.docx]

**Supplementary Materials**

**Human health risk assessment of potentially toxic elements (PTEs) in the breast milk consumed by infants in Western Iran**

**Table S1** The body weight and daily consumption of breast milk based on various infants 'age

| Age (Month) | Weight (Kg) | | DCBM (mL/d) |
| --- | --- | --- | --- |
|  | Boy | Girl |  |
| 1 | 4 | 4.3 | 650.5 |
| 2 | 5.6 | 5.3 | 843 |
| 3 | 6.4 | 6 | 916.5 |
| 4-6 | 7.5 | 7.05 | 887 |
| 7 | 8.3 | 7.9 | 916.5 |
| 8 | 8.6 | 8.2 | 916.5 |
| 9 | 8.9 | 8.5 | 916.5 |
| 10-12 | 9.35 | 9 | 798.5 |

DCBM: Daily consumption of breast milk

**Table S2** The heavy metals concentration of drinking water

| Heavy metals | Descriptive parameters (µg L^-1^) | | | | Standards | | |
| --- | --- | --- | --- | --- | --- | --- | --- |
|  | Mean | SD | Min | Max | WHO | EPA | IRAN |
| As | 7.9 | 4.9 | 2.2 | 16.3 | 10 | 10 | 10 |
| Cd | 0.1 | 0.07 | 0.1 | 0.3 | 3 | 5 | 3 |
| Hg | 0.5 | 0.2 | 0.2 | 0.9 | 6 | 2 | 6 |
| Pb | 3.3 | 1.4 | 1.1 | 5.9 | 10 | 15 | 10 |
| Cr | 6.2 | 2.9 | 2.3 | 11.2 | 50 | 100 | 50 |
| Ni | 16.0 | 6.0 | 10.1 | 26.4 | 70 | 20 | 70 |

**Figure S1** Box-plot of heavy metal concentration levels in drinking water of participants. The bold points in orange and blue color represent EPA and WHO guideline values.
